# Quality and Shelf-Life Evaluation of Fresh Beef Stored in Smart Packaging

**DOI:** 10.3390/foods12020396

**Published:** 2023-01-13

**Authors:** Andi Dirpan, Serli Hatul Hidayat

**Affiliations:** 1 Department of Agricultural Technology, Faculty of Agriculture, Hasanuddin University, Makassar 90245, Indonesia; 2 Center of Excellence in Science and Technology on Food Product Diversification, Makassar 90245, Indonesia; 3 Research Group for Post-Harvest Technology and Biotechnology, Makassar 90245, Indonesia

**Keywords:** garlic extract, packaging indicators, packaged beef

## Abstract

Beef is a perishable food product susceptible to deterioration due to microbial growth. Therefore, this study aimed to ascertain how active and intelligent packaging performs by tracking the change in the quality of fresh beef stored at low temperatures. The intelligent packaging method employed indicators with solutions of Bromo Phenol Blue (BPB) and Phenol Red (PR) to monitor the change in beef quality. Additionally, active packaging used garlic extract with various concentrations at 0%, 15%, and 20% to maintain the quality of beef packaged at 10 °C temperatures. The findings illustrated that a packaging indicator label can be implemented to monitor the change in the quality of fresh beef stored at 10 °C temperatures. This was signified by a change in the indicator color from dark yellow to orange and red, fading to purple. Meanwhile, observations on active packaging demonstrated that 15% and 20% of garlic extract were the most effective approaches for preserving beef quality. The correlation level of indicator label color analysis and the effectiveness of active packaging with all beef spoilage metrics demonstrated a positive correlation in preserving quality and identifying the degree of beef damage. Therefore, these active and intelligent packaging indicators can be applied to monitor and retain the quality of packaged beef.

## 1. Introduction

Beef is categorized as a perishable food due to its restricted shelf-life and susceptibility to quality degradation during storage. The high protein, water, nitrogen, and fat contents in the beef act as favorable media, making it prone to the growth of spoilage microorganisms [1,2,3].

The rapid decline in beef quality jeopardizes consumer health and results in distributors’ losses [4]. Indispensable factors, namely marketing and packaging conditions, catalyzes the decline in quality. Poor conditions, such as open marketing spaces without packaging and serving in unsanitary places with high air temperatures, consequently accelerated pathogenic microbial contamination, characterized by changes in color, texture, and flavor, leading to spoilage.

In order to extend the shelf-life and monitor the quality of beef products, an innovative approach of smart packaging, a combination of intelligent and active packaging, can be introduced. This packaging is capable of not only monitoring and alerting to a change in quality based on variations of the indicator color but also releasing an active substance that can prevent microbial contamination. Implementation of this practice will prove to be beneficial for both distributors and sellers by reducing losses, adjusting market prices based on intelligent packaging information [5,6,7,8,9,10], and protecting the products from spoilage from microbial contamination. The growth of microorganisms can be inhibited by applying active packaging containing antimicrobials, such as garlic essential oil [11]. Garlic extract contains allicin compounds that have an anti-bacterial function [12,13,14], which can inhibit a change in the color, aroma, and taste of beef [9,15,16,17]. Nevertheless, garlic (*Allium sativum* L.) possesses a strong taste and aroma due to its high phenolic and sulfuric content [18]. Organosulfur plays a significant role in the development of flavor and scent, including allicin, diallyl sulfide, and diallyl trisulfide [19]. The strong taste and aroma alter the food product, which also has an adverse effect on the product’s organoleptic acceptance [20]. Therefore, it is essential to use the proper quantity of garlic, and as little as possible, to prevent sensory changes to food products.

The application of active packaging has been studied by several researchers, including Wiastuti et al., who applied ginger oleoresin in various concentrations in the production of active packaging, and Iriani et al., who added garlic extract as an active substance applied to fresh beef product [2,21].

Similarly, smart (a combination of intelligent and active) packaging has proven to be comparatively more effective in providing information about the state of packaged food, reducing the wastage of products, and protecting from spoilage by microbial contamination [22,23,24,25,26,27]. Since intelligent packaging is only effective in maintaining the shelf life of packaged products, a combination with active packaging is essential. Therefore, this study aimed to emphasize the effectiveness of applying both intelligent and active packaging on beef stored at 10 °C temperatures, which was evaluated by observing changes in beef, such as the pH values, Total Bacterial Count (TBC), Total Volatile Basic Nitrogen (TVBN), and Thio Barbituric Acid (TBA), as well as indicator sensitivity to change in addition to the deterioration in beef quality. This study assessed the freshness of beef packaged in smart packaging based on the indicator’s shelf life and color change when stored at 10 °C.

## 2. Materials and Methods

### 2.1. Materials

The materials used were fresh beef (Makassar, Indonesia), filter paper (Whatman^TM^) (Merck, Darmstadt, Germany), Bromothymol Blue (BTB) (Merck, Darmstadt, Germany, CAS No:76-59-5), Phenol Red (PR) (Merck, Darmstadt, Germany, CAS No. 34487-61-1), garlic, 96% alcohol (Merck, Darmstadt, Germany), tapioca starch (rose brand, Jakarta Selatan, Indonesia), acetic acid (Sigma Aldrich, St. Louis, MO, USA), food grade chitosan powder (Sigma Aldrich, St. Louis, MO, USA), tween^®^ 80 (Sigma Aldrich, St. Louis, MO, USA, CAS No.9005-65-6), hydrochloric acid (HCL) (Merck, Darmstadt, Germany), 2-Thiobarbituric acid (TBA) (Merck, Darmstadt, Germany), glacial acetic acid (Brenntag Inc., Essen, Germany), trichloroacetic acid (TCA) (Merck, Darmstadt, Germany), K_2_CO_3_ (Merck, Darmstadt, Germany), and nutrient agar (Merck, Darmstadt, Germany).

### 2.2. Stages

#### 2.2.1. Preparation of Intelligent and Active Packaging Indicators

The intelligent packaging indicator was prepared using 2 × 4 cm filter paper (Whatman ^TM^) soaked in 10 mL of a solution of BTB and PR at a pH of 5 with a ratio of (1:1) for 24 h at room temperature (28 ± 2 °C). The indicator was washed with running water to remove the unbound solution in the filter paper membrane. Subsequently, it was dried with a hair dryer (Philips) and stored in a closed container [28,29].

Active packaging was assembled using 110 mm filter paper (Whatman ^TM^) of approximately 15 g that was soaked for 24 h in 20 mL of distilled water. The filter paper was crushed to form a pulp, which was added with tapioca starch solution (rose brand) 30% (*w*/*w*), 100 mL of acetic acid (Sigma Aldrich, St. Louis, MO, USA) 1% (*w*/*v*), and food-grade chitosan powder (Sigma Aldrich, St. Louis, MO, USA) 0.45% (*w*/*w*). Garlic extracts at 0%, 15%, and 20% (*w*/*w*) were added to 50 mL of distilled water and put into the pulp. After becoming homogeneous, 0.205 g of tween^®^ 80 (Sigma Aldrich, St. Louis, MO, USA) was poured and stirred until an emulsion was formed. Further, the solution was poured onto a 20 cm × 30 cm tray to form a wet paper sheet and dried at 30 °C for 48 h [21].

#### 2.2.2. Application of Intelligent and Active Packaging on Fresh Beef

**Figure 1:**
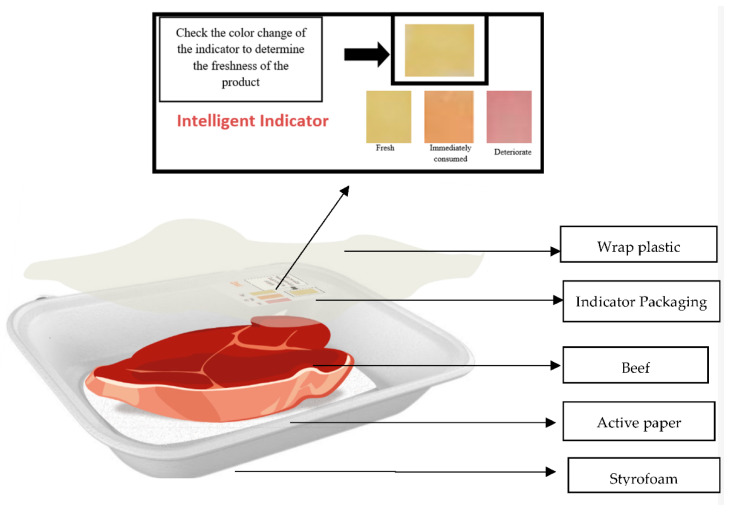
Smart packaging implementation design on beef.

Approximately 250 g of fresh untreated beef (*tenderloin*) with a postmortem pH of 5.4 obtained from the Tamangapa Raya Makassar Slaughterhouse, Makassar, Indonesia, was packed in Styrofoam (1.05 g/cm^3^) and covered with Low-Density Polyethylene (LDPE) (0.9 g/cm^3^) plastic. Furthermore, the indicator label was placed inside the packaging and affixed to the surface of the LDPE instead of directly touching the product. The sample was stored at 10 °C and observed for 0, 3, 6, 9, 12, and 15 days. The indicators were compared with the result of each rot test analysis for the beef (pH, TVBN, TBC, and TBA). The smart packaging implementation design is shown in Figure 1.

### 2.3. Observation Parameters

#### 2.3.1. Analysis of Intelligent Packaging Indicator Color Change

The indicator color was measured using a digital colorimeter test (T-135) for the values of L (brightness), a (red-green mixed chromatic color), and b (blue-yellow chromatic color) by attaching the colorimeter sensor to the label. Indicator color quantification was carried out using a digital camera to capture the indicator image visually. Images were taken under identical light settings from a distance of ±20 cm and placed in a box [29,30]. The a and b values obtained were converted to Hue with the formula:

◦Hue = tan^−1^ (b/a) (1)

Descriptions:

◦Hue: parameters for color range

a = red-green mixed chromatic color

b = blue-yellow chromatic color

#### 2.3.2. Thio Barbituric Acid (TBA) Value

A sample of 3 g was added to 50 mL of distilled water and mashed for 2 min. The sample was put in a 1000 mL distillation flask and washed with 48.5 mL distilled water. Further, 1.5 mL of HCl was added, and the distillate flask was heated for 10 min to obtain 50 mL. The resulting distillate was filtered and added to 5 mL of TBA reagent (0.02 M TBA solution in 90% glacial acetic acid). The dissolution process was accelerated by heating the distillate in a water bath for 35 min. The obtained product was cooled with cold water, and the optical density was measured with a spectrophotometer at a wavelength of 528 nm with the blank solution as the zero point [31].

#### 2.3.3. Total Volatile Basic Nitrogen (TVBN) Value

The beef sample was weighed to 10 g ± 0.1 g, added to 30 mL of 7% TCA solution, blended, and filtered until the filtrate was obtained. A total of 1 mL of boric acid solution was put into the ‘inner chamber’ of the Conway cup, closed, and placed in a position almost covering the cup. The filtrate was put into the outer chamber, and 1 mL of saturated K_2_CO_3_ solution was added; hence, the filtrate and K_2_CO_3_ were not mixed. The cup was immediately closed and moved around to mix the two liquids in the outer chamber. The blank solution was carried out with the same procedure, but the filtrate was replaced with a 7% TCA solution and stored at 37 °C for 2 h. Further, the boric acid solution and Conway dish containing the blank and the filtrate were titrated with 0.01 N HCl until they turned pink. The determination of the TVBN value was calculated using the following formula:

(2) $$\mathrm{Total}\;\mathrm{TVBN}\;(\mathrm{mg}/100\;g)=\frac{V_{c}{\;-\;V}_{b}-N\;\times \;f_{p}\;\times \;100}{w}$$

Description:

V_c_ = volume of HCl solution in sample titration

V_b_ = volume of HCl solution in blank titration

N = normality of HCl solution

w = sample weight (g)

14.007 = atomic weight of nitrogen

f_p_ = dilution factor

#### 2.3.4. Total Bacterial Count (TBC)

The determination of TBC was carried out using the cup count method described in the Indonesian National Standard (SNI; 2897:2008). Here, 1 g of sample was diluted in 9 mL of sterile physiological solution (0.85% NaCl) equivalent to (10^−1^). The dilution process was carried out until it reached a dilution of 10^−6^. Further, the cultivation process with the pouring method was 1 mL of samples from dilutions of 10^−4^, 10^−5^, and 10^−6^ poured into different sterile petri dishes in duplicate. Then, 15 mL of NA media was added and incubated at 30 °C for 48 h. Subsequently, the TBC calculation was calculated using the following formula:

(3) $$N=\frac{\sum C}{\left[\left({1\;\times \;n}_{1}\right)+\left(0{.1\;\times \;n}_{2}\right)+\ldots \right]\;\times \;(D)}$$

where:

N = number of colonies per mL/per gram of product

ΣC = total number of colonies counted

n_1_ = number of petri dishes in the first dilution

n_2_ = number of petri dishes in the second dilution

D = first dilution counted

#### 2.3.5. pH

The pH measurement assessed the initial and final pH during the storage of fresh beef. The meter used was a digital Horiba Laquatwin Compact pH Meter P-33 with an accuracy of 0.01%. Approximately 0.5 g of sample was placed on the surface of the pH meter sensor until the value was displayed on the screen.

### 2.4. Data Analysis

Each analysis was conducted with three replications, processed using the Statistical Package for the Social Sciences (SPSS) analysis of variance (ANOVA) data, and further tested with Duncan’s test. Furthermore, significant treatment differences were indicated by *p* < 0.05.

## 3. Results and Discussion

### 3.1. Intelligent Packaging Indicator Color Change

**Figure 2:**
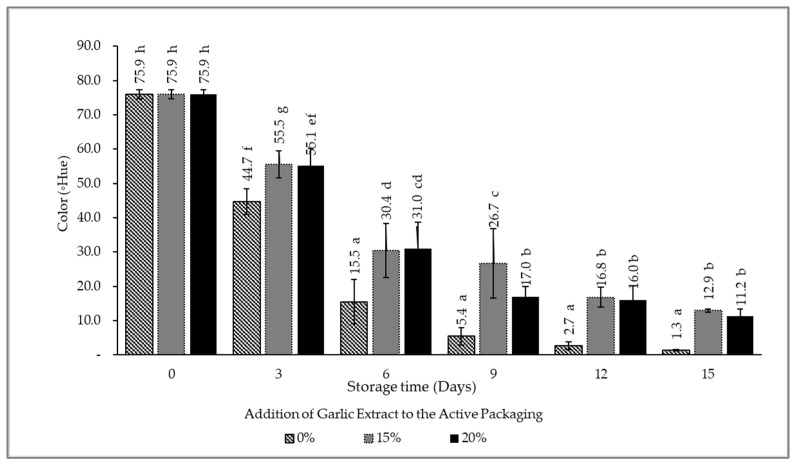
Graph for the Hue value of the indicator label during storage. The mean value followed by various letters indicates a significant difference (*p*-value < 0.05).

**Figure 3:**
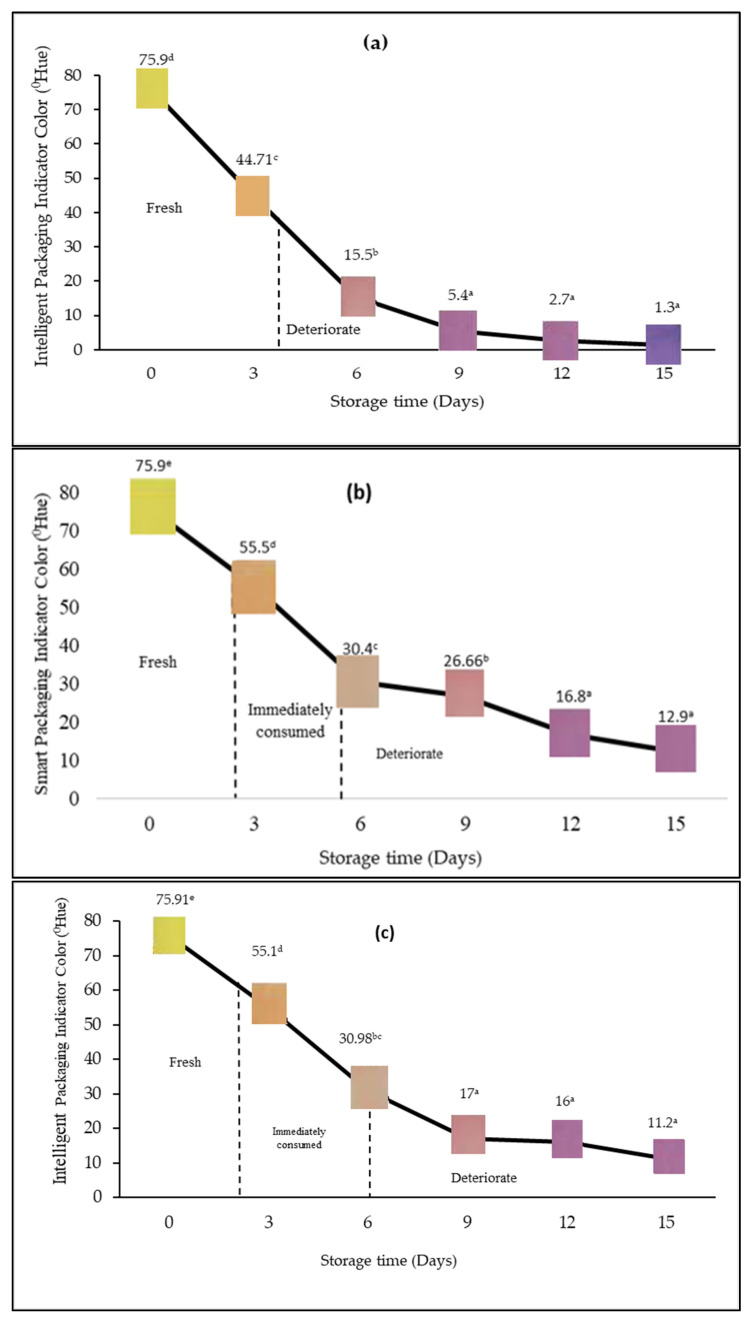
Change in the color of the intelligent packaging indicator for the packaged product. (**a**) Indicator label with the addition of 0% garlic extract, (**b**) indicator label with the addition of 15% garlic extract, and (**c**) indicator label with the addition of 20% garlic extract. The mean value followed by various letters indicates a significant difference (*p* < 0.05).

Intelligent packaging was equipped with an indicator label as a source of information regarding the product’s condition. The indicator’s color change was a sign of the response to the quality of the packaged beef. The analysis of color change was carried out to determine the phase of the intelligent packaging indicator using PR and BTB (1:1). The measurement was carried out using a digital color meter (T-135) that showed the values of l, a, and b as international measurement standards published by the Hunterlab Association Laboratory. The values of l, a, and b were converted to 0Hue, and the results are depicted in Figure 2 and Figure 3.

Figure 2 depicts a comparative analysis of color variations that occur at each concentration of garlic extract presented in one graph, and Figure 3a–c provides insight into the color gradations that occur in intelligent packaging indicators during storage at each concentration of garlic extract. Distinct phases of indicator color variation indicate the quality condition of the product, namely the phase I yellow indicator indicates that the meat is still fresh, the phase II orange color indicates that the meat must be consumed immediately, and the phase III purple color indicates that the meat is not suitable for consumption. The color change on the intelligent packaging indicator label signifies product deterioration and its close association with an increased number of microbes during storage. Microbes catalyze the decomposition of nutritional components to produce volatile base compounds. These compounds lead to an increase in the total volatile basic nitrogen value and further elevate the product’s pH. The presence of volatile base compounds in the packaging, when absorbed by the indicator label, eventually changes its color from dark yellow to purple. According to De Meyer, the BTB solution in a protonated condition will produce a yellow color and experience a blue or turquoise change. Meanwhile, Melati stated that the PR solution would experience a color change to yellow and red from acidic to alkaline conditions [32,33]. Since this study combined BTB and PR, it resulted in yellow acidic conditions and purple alkaline conditions, which is a combination of blue and red colors.

Figure 2 demonstrates that the ◦Hue during storage for all treatments was diminished, signifying the quality degradation of the product. Based on observations, it can be determined that the addition of garlic extract both at 15% and 20% reduced the indicator’s color change compared to the control (no or 0% garlic extract addition). However, the graph also exhibits that, in general, there is no significant difference between adding 15% and 20% of the garlic extract.

As displayed in Figure 3a, in the absence of garlic extract, the indicator label exhibited a purple color after three days of storage, whereas with the addition of garlic extract at 15% and 20% (Figure 3b,c), the indicator label changed to purple after six days of storage, establishing that the application of garlic extract adds three supplementary days of shelf life to the beef product in this study. Garlic extracts (15% and 20%) contain allicin compounds that function as an anti-bacterial substance inhibiting bacterial growth [12,13,14], which hinders the decomposition of the nutritional components that produce volatile base compounds and bind to the indicator.

### 3.2. Thio Barbituric Acid (TBA) Value

**Figure 4:**
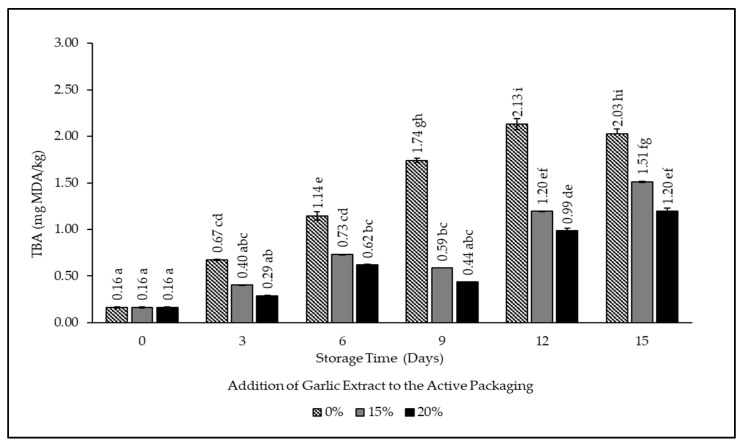
TBA value of beef packaged using intelligent and active packaging with the addition of garlic extract. The mean value followed by various letters indicates a significant difference (*p* < 0.05).

The TBA value calculates the malondialdehyde (MDA) value. MDA is an aldehyde compound resulting from lipid oxidation in beef, which not only reduces the quality but also gives a rancid aroma to the product [34,35]. The TBA test is determined based on the formation of a pink pigment due to a condensation reaction between two TBA and one malonaldehyde molecule. The saturated fat in beef tissue is directly proportional to the malonaldehyde formed [36]. The TBA value of beef stored in intelligent and active packaging is shown in Figure 4.

Based on Figure 4, the TBA value of all samples increases significantly during fifteen days of storage. The initial value of beef was 0.16 mg MDA/kg and continued to increase until day fifteen. The TBA value of the sample without the addition of garlic extract (0%) experienced a significant increase (*p* < 0.05) compared to those with 15% and 20% garlic extract. According to Behrozz et al., the acceptable threshold for TBA in beef is 1 mg MDA/kg [37]. During the fifteen days of storage, the sample without the garlic extract had a TBA value of 1.14 mg/MDA/kg on day three. Meanwhile, the sample with the addition of 15% and 20% garlic extract had a TBA concentration close to the threshold on day six, which were 0.73 and 0.62 mg/MDA/kg, respectively. After day nine, the packaged meat had passed the standard TBA value. The difference in the length of time of TBA increase in the 15% and 20% treatments is due to the role of bactericidal and antioxidant properties found in garlic that can inhibit the oxidation of unsaturated fatty acids. This is in accordance with the statement of Purnamasari et al. that compounds that are bactericidal and antioxidant in nature can inhibit the formation of unsaturated fats that initiates the formation of the rancidity process in food ingredients [34].

### 3.3. Total Volatile Basic Nitrogen (TVBN) Value

**Figure 5:**
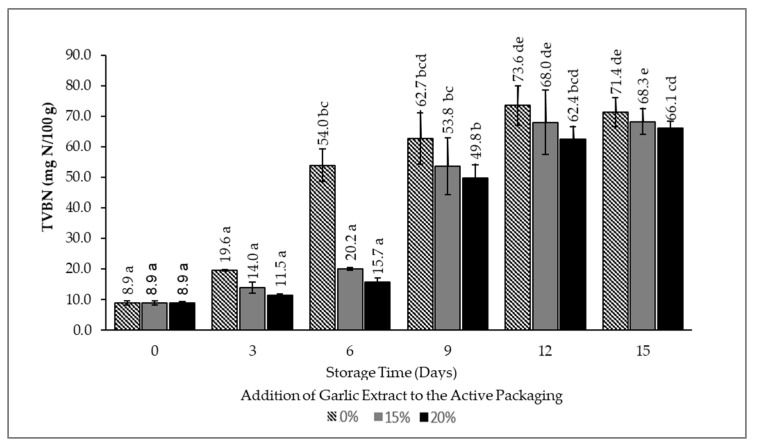
TVBN value of beef packaged using intelligent and active packaging with the addition of garlic extract. The mean value followed by various letters indicates a significant difference (*p* < 0.05).

TVBN is one of the tests used to measure nitrogen compounds, or biogenic amines, due to bacterial activity on beef spoilage [35,38,39]. The increase in the TVBN value is due to bacteria’s breakdown of protein into peptide bonds and amino acids and then into simpler nitrogen compounds, such as trimethylamine, dimethylamine, and ammonia. Furthermore, the protein breakdown is volatile, resulting in a foul odor. The TVBN value is directly proportional to bacterial activity in beef spoilage. According to Alizadeh-Sani et al., the acceptable threshold value for TVBN is 25 mg N/100 g [40]. The TVBN value obtained in meat storage at 10 °C with various concentrations of garlic extract can be seen in Figure 5.

Figure 5 depicts that beef stored at 10 °C and wrapped using smart packaging experienced an increase in the TVBN value during storage. The TVBN value on day zero was 8.9 mg N/100 g, indicating that the beef was in fresh or good condition. However, the value increased significantly in each treatment during storage. The sample without garlic extract exhibited an increased accumulation of TVBN, followed by those with 15% and 20% of the extract. The TVBN value of samples packaged without the addition of garlic extract crossed the threshold after day three, while the TVBN value of samples with the addition of 15% and 20% of garlic extract surpassed the threshold after six days of storage with no significant difference between the use of 15% and 20% of garlic extract. It can be concluded that the application of garlic extract (both at 15% and 20%) can prevent the production of TVBN. This is due to the presence of allicin in garlic extract that possesses antimicrobial characteristics that prevent the growth of microorganisms, and thereby TVBN accumulation, in the beef product. The antimicrobial ingredients in the garlic extract work by attacking DNA, synthesizing proteins, and inhibiting the bacterial RNA system. Another study also reported that the active components in the extract can impede the formation of spoilage microbes in food products [41,42].

### 3.4. Total Bacterial Count (TBC) Value

**Figure 6:**
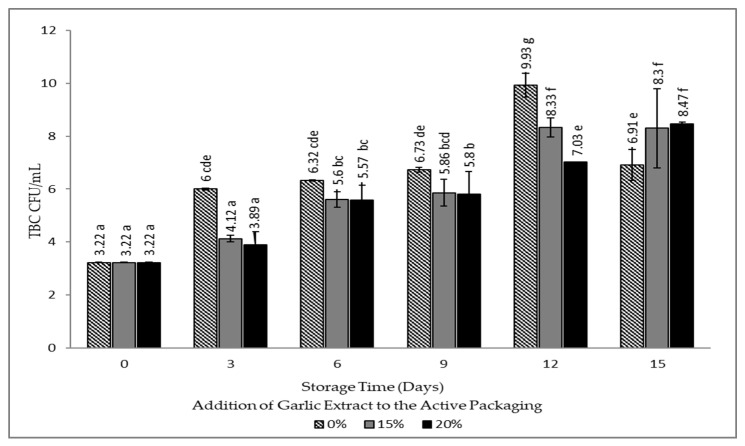
Graph for TBC value of beef packaged using intelligent and active packaging with the addition of garlic extract. The mean value followed by various letters indicates a significant difference (*p* < 0.05).

The protein, water, nitrogen, and fat contents of beef serve as a favorable media increasing its susceptibility to the growth of spoilage microorganisms [1,2]. The TBC analysis was carried out to determine the effect of active packaging on the level of bacterial contamination in the packaged beef product, which can affect the freshness level based on the number of contaminants. The requirements for carcass and beef regulated in Standard Nasional Indonesia (SNI 3932:2008) illustrate that the limit for TBC microbes is 1 *×* 10^6^ or 6 CFU/mL. The test results of the TBC value are depicted in Figure 6.

Figure 6 shows an increase in the TBC value of fresh meat storage at 10 °C. The TBC value of meat with the treatment and without the addition of garlic extract (0%) exceeded the TBC standard on day three, whereas the treatment with the addition of 15% and 20% garlic extract crossed the TBC threshold after day nine. This indicates that the addition of 15% and 20% garlic extract to meat packaging has an effect in inhibiting microbial growth and extending the shelf life of meat compared to meat samples without the addition of garlic extract. The difference in the duration of the increase in the TBC value is due to the addition of garlic extract to activated paper, which can inhibit the growth of gram-negative and gram-positive bacteria due to the presence of antimicrobial compounds in the form of allicin. Garlic extract applied to activated paper will diffuse to the entire surface of the beef so that it can inhibit bacterial growth. The process of inhibiting bacteria involves total inhibition of RNA synthesis and partial inhibition of DNA and pro-protein synthesis. Allicin blocks bacterial enzymes belonging to thiol groups, consequently impeding bacterial growth. This is in accordance with the statement of Boboye et al. that the mechanism of garlic inhibits bacterial growth by comprehensively inhibiting RNA, DNA, and protein synthesis of bacteria [43,44].

### 3.5. pH

**Figure 7:**
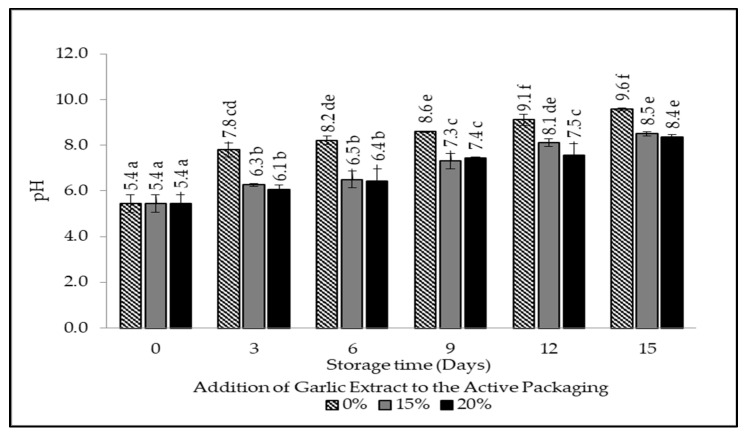
The pH value of beef packaged using intelligent and active packaging with the addition of garlic extract. The mean value followed by various letters indicates a significant difference (*p* < 0.05).

The degree of acidity in food products expresses the level of acidity or basicity of a substance or object [45]. The pH of live beef ranges from 7.0–7.2; however, it quickly declines after cattle are slaughtered. This is caused by the anaerobic glycolysis process due to the cessation of oxygen delivery to the muscles. In glycolysis, glycogen reserves are converted into lactic acid, thereby reducing the pH. This process ceases with the depletion of glycogen levels. The pH measurement is considered one method to determine the freshness level of beef. In this study, a comparative analysis was conducted to determine the effect of garlic extract on the pH value of packaged beef, as shown in Figure 7.

Figure 7 exhibits an increase in pH values in each treatment during storage due to protein degradation by microbes resulting in ammonia production. Ammonia deprotonates water to produce ammonium ions and hydroxide OH- [27]. Samples with treatment and without the addition of garlic extract experienced a faster increase in the pH value, namely from a pH value of 5 to a pH of 7 before day three. In comparison, samples with both 15% and 20% additions of garlic extract experienced an increase in the pH value of 5 to 7 after day nine. The difference in the increase in the pH value is due to the presence of the garlic extract added to the active packaging, which contains allicin compounds that act as antimicrobial agents, especially to spoilage microbes, that can hinder the protein degradation process [46,47,48]. The mechanism of allicin in inhibiting bacterial growth by increasing the permeability of the phospholipid bilayer does not occur due to the absence of the production of the amino acids and proteins that cause SH groups (sulfhydryl and disulfide) to be destroyed on the amino acids cystine and cysteine. The destroyed SH groups inhibit the synthesis of the protease enzymes that damage the cytoplasmic membrane of the bacterial wall and interfere with protein and nucleic acid metabolism resulting in bacterial proliferation [13,14,49].

### 3.6. Correlation between Indicator Label Sensor Response with Various Parameters of Quality Deterioration in Packaged Fresh Beef

**Figure 8:**
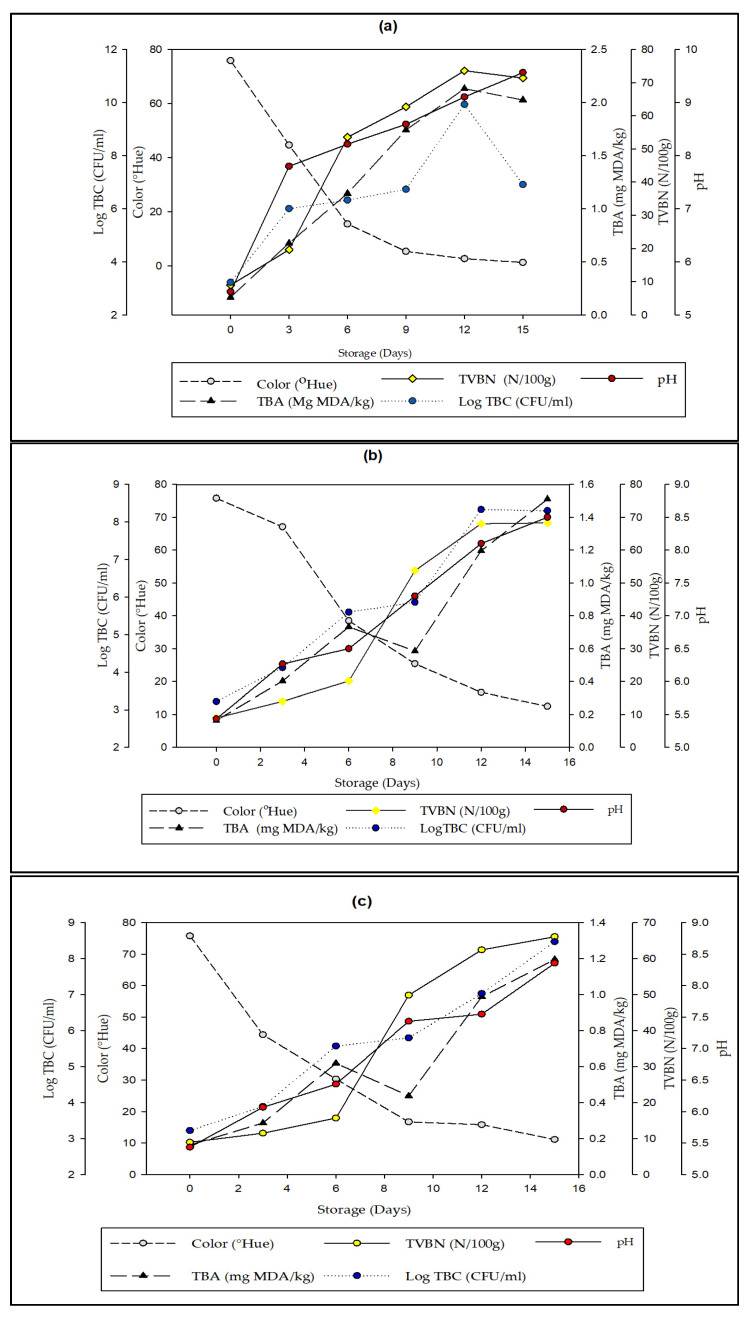
Correlation between indicator label sensor response with various parameters of quality deterioration in packaged fresh beef (**a**) 0%, (**b**) 15%, and (**c**) 20%.

Correlation of the response to the color change of intelligent and active indicator labels with each test parameter on beef, such as TVBN, TBC, and pH, was carried out to determine the deterioration of beef quality and obtain synchronization. The comparison of the color change in the intelligent indicator label with the test parameters of beef quality deterioration can be seen in Figure 8.

Based on Figure 8, beef stored without garlic extract (0%) experienced a rapid increase in the TVBN, TBC, TBA, and pH values compared to beef with garlic extract (15% and 20%), in line with the decrease in the indicator color value of intelligent packaging. The difference in the parameter value for the beef quality deterioration of each treatment is due to the garlic extract in the active packaging [50,51].

Storage at cold temperatures also affects the shelf life of beef. In this study, beef without garlic extract diminished in quality and was unfit for consumption on day three. Meanwhile, beef with 15% and 20% garlic extract deteriorated and was found to be unfit for consumption on day nine. This indicates that storage at cold temperatures with garlic extract can extend the shelf life of beef due to the nature of allicin. This is consistent with Suradi, who found that beef stored at refrigerator temperatures (5 °C) without active components had a shelf life based on the TVBN value of 195 h 43 min. The intelligent indicator label using Whatman paper no.1 and the BTB + PR (pH 5.00) indicator can be applied as an indicator to assess the freshness of the meat with a color change from yellow (initial) to faded red and purple (final). This can help consumers to determine freshness without touching or opening the beef packaging [52].

The use of 15 and 20% garlic extract did not show any appreciable differences in the majority of the parameters examined, including TBC, pH, and TVBN. This led to the conclusion that both surveys have an equal impact on preserving the quality of packaged beef. In light of organoleptic considerations, we advise applying 15% garlic extract.

## 4. Conclusions

Based on the results, the intelligent packaging indicator labels effectively detect the quality of beef stored at 10 °C. The color change profile of the intelligent indicator label is identified by dark yellow, showing the beef is still fresh, orange, which should be consumed immediately, and faded red to purple colors, which is unsuitable for consumption. Moreover, garlic extract functions as an antimicrobial substance when added to the packaging and can suppress the growth of spoilage bacteria in beef with concentrations of both 15% and 20%, with no significant effects in most parameters tested. Therefore, it is suggested to apply 15% garlic extract. Implementation of active and intelligent packaging indicators will prove to be beneficial to monitor and retain the quality of packaged beef.

## Data Availability

Available data are presented in the manuscript.
